# The Engineering Side of Hospital Work

**Published:** 1905-04-15

**Authors:** 


### HOSPITAL ADMINISTRATION.

### CONSTRUCTION AND ECONOMICS.

### THE ENGINEERING SIDE OF HOSPITAL WORK.

*Continued from page 34.*

IX.—LAUNDRIES. DRYING THE CLOTHES.

As explained in the previous article, the process of drying is divided into two operations, corresponding to the old processes of wringing and drying in the sun. Nearly all the water is first squeezed out of the clothes by one of the following apparatus. For what washerwomen would call a small wash—a small number of any particular article—is the well-known wringing machine, consisting of two vulcanised rubber rollers, fixed close together in a frame, usually in a vertical position, with wheel gearing attached, so that motion can be given to both rollers by a handle at the side, or by power from any convenient source. The clothes are passed individually between the two rollers, which are arranged to give slightly, to allow the clothes to pass, but which squeeze them as they pass, and extract nearly all the moisture from them. But where a large number of clothes has to be dealt with, the process by this machine, efficient as it is, is too slow. Hence apparatus has been worked out, wringing large numbers of things at the same time. By far the commonest kind of machine in use for this purpose is of the type called, rather erroneously again, the "Hydro." Its proper name is the centrifugal machine, because it extracts the water from the clothes by the application of centrifugal force. The apparatus consists always of a cylindrical vessel, fixed vertically with a basket, also in the form of a cylinder, carried on the inside. Both the outer cylinder and the basket are uncovered at the top, though in some cases a cover is provided for the whole apparatus, which is put on, when the clothes are in the machine, to keep out the dirt. The basket is made of either galvanised iron wire, copper wire, or perforated copper, brass, or galvanised iron. The basket is suspended on a vertical axle, upon which it revolves, and motion is communicated to it, either from above or below. The clothes to be dried are placed in the basket, which is then caused to revolve rapidly. The centrifugal force created by the motion of the basket causes the clothes to press against the periphery of the basket, and in so doing the water that is in them is squeezed out into the outer cylinder, whence it runs away to the drains. The force exerted by the clothes upon themselves in this process is found from the formula, Force exerted in lbs., forcing the clothes out = the weight of the clothes, multiplied by the square of the rate at which they are travelling round (the circumferential velocity of the cage practically) and divided by the radius of the circle in which they are moving, the radius of gyration as it is called, and by the force of gravity, taken as 32·2. The velocity of gyration, and the force of gravity are measured in feet per second. With a machine running at a high speed, the force applied to the clothes is considerable. Motion is given to the cage, usually by a horizontally running bevelled friction pulley, bearing against a vertically running bevelled friction pulley, the first pulley receiving motion from a horizontal shaft that may be carried either above the machine or below. The machine may be driven by a separate small engine, by an electric motor, or by any convenient source of power, all that is necessary being that rotary motion shall be given to the cage, and that it shall be easy and safe to start and stop the apparatus at will. After the centrifugal machine has run for the time necessary according to the clothes that are being operated upon they are removed to the drying-closets or drying-room.

Drying Rooms and Drying Closets.

Drying rooms and drying closets are all arranged on one principle. Air has a certain capacity for absorbing moisture, depending upon its temperature. At a temperature of 40° F. each cubic foot of air will absorb 3 grains of water vapour; at 60° F. the quantity rises to $5\frac{3}{4}$; at 80° it is 11 grains, and at 100° it will carry 20 grains. This ability of atmospheric air to carry moisture, differing in quantity according to its temperature, is at the bottom of a good many things in common life—rain for instance. In the present case it affords the laundry engineer a simple method of drying clothes quickly, and well. The clothes to be dried are placed on horses, or similar arrangements, and a continuous stream of warm air is caused to pass over them. Each cubic foot, or cubic inch of air, as it passes absorbs a small portion of the moisture remaining in the clothes, causing it to form into vapour, and the continual passage of the stream of air dries the clothes in a comparatively short time. It will be understood that the mere presence of warm air in the drying closets would not be sufficient to dry the clothes under practical conditions. Apart from the fact that a large quantity of air would be required to absorb the whole of the moisture in any quantity of clothes, such as have to be dealt with in hospitals, the effect of the evaporation of the water, as explained in the article on Cold Storage, is to cool the air. When water forms into vapour, it will be remembered a certain number of heat units are demanded, in order that it may be able to do so; and the heat demanded is taken from every object at a higher temperature than that assumed by the evaporating water at the moment, that is from every object with which the water is brought into contact; with the result that the temperature of all of them is lowered. It will be remembered that this forms a valuable method of cooling water that has already done duty in a condenser, as well as in one form of the condenser itself. It also cools the air to which the vapour is added, with the result that the ability of that particular quantity of air to hold moisture is lowered, and the object sought would not be attained unless a large quantity of air was in operation. The difficulty is overcome by the constant stream of air passing over the clothes. The apparatus performs the work that a good drying wind, as it is termed, does, but with the advantage that the supply of air, and the temperature to which the air is raised, are under the control of the engineer. The arrangement usually employed is as follows. The clothes are spread upon a number of horses, corresponding roughly to the old-time clothes-horse. The horses consist of a number of galvanised iron rods fixed between pairs of vertical cast-iron plates, one of each pair forming the back and the other the front of the apparatus. The horses all push into a large brick-built chamber, the backs forming one continuous iron wall, and the fronts, which are made more or less ornamental, and which carry the handles by which the horses are withdrawn, close in together, so that they also form a continuous wall, extending from the roof of the chamber to the floor. The complete horses are usually fixed on rollers, which run on rails provided for them in the floor of the chamber and that of the room into which the horses are withdrawn. A variation of the rails on the floor is an arrangement of rails above, running back a sufficient distance outside the closets to enable the horses to be completely withdrawn, and with rollers fixed on to the upper side of the fronts and backs of the horses. The horses are sometimes made by Messrs. Isaac Braithwaite and Son, of Kendal, all of wood. Another very pretty variation of the drying arrangements, made by the same firm, especially for starched things, is their "Ibis" Automatic Conveyor Drying Room. The drying chamber is constructed on the usual lines, but in place of horses arranged to draw in and out, a frame, carrying a conveying apparatus, extends some distance in front of the drying chamber. On the conveyor are a number of hooks, and the laundry girls hang each piece to be dried upon one of the hooks. The conveyor, being continually in motion, carries the articles into the drying room, keeps them there a sufficient length of time to completely dry them, then carries them out on the opposite side and automatically drops them into baskets arranged to receive them. The conveyor principle has not, the writer believes, been much used yet in laundry work, but it is largely used in the biscuit and confectionery manufactures, where the conditions are somewhat similar. In another form of the closet, used where room is scarce, the horses do not pull out, and are constructed in radial form. Each horse consists of a number of galvanised iron or wooden bars, fixed radially in a vertical pillar, so that they can be turned round by the attendant, who, in this case, has to go into the closet.

